# The Stress-Dependent Activation Parameters for Dislocation Nucleation in Molybdenum Nanoparticles

**DOI:** 10.1038/s41598-018-21868-y

**Published:** 2018-03-02

**Authors:** Doron Chachamovitz, Dan Mordehai

**Affiliations:** Mechanical Engineering, Technion, 32000 Israel

## Abstract

Many specimens at the nanoscale are pristine of dislocations, line defects which are the main carriers of plasticity. As a result, they exhibit extremely high strengths which are dislocation-nucleation controlled. Since nucleation is a thermally activated process, it is essential to quantify the stress-dependent activation parameters for dislocation nucleation in order to study the strength of specimens at the nanoscale and its distribution. In this work, we calculate the strength of Mo nanoparticles in molecular dynamics simulations and we propose a method to extract the activation free-energy barrier for dislocation nucleation from the distribution of the results. We show that by deforming the nanoparticles at a constant strain rate, their strength distribution can be approximated by a normal distribution, from which the activation volumes at different stresses and temperatures are calculated directly. We found that the activation energy dependency on the stress near spontaneous nucleation conditions obeys a power-law with a critical exponent of approximately 3/2, which is in accordance with critical exponents found in other thermally activated processes but never for dislocation nucleation. Additionally, significant activation entropies were calculated. Finally, we generalize the approach to calculate the activation parameters for other driving-force dependent thermally activated processes.

## Introduction

Using pristine (defect-free) specimens as the basic building blocks of nanomechanical devices has become increasingly popular. The strength of these specimens is much higher than their bulk counterparts since dislocations, the line defects within the lattice structure that are the main carriers of plasticity, must be nucleated into the perfect lattice in order to deform them plastically. Generally, nucleation is a probabilistic process in which a system escapes from a local equilibrium by overcoming a free-energy barrier. Many mechanisms at the nanoscale involve the process of overcoming energy barriers, such as diffusion^1^, the plastic deformation of metallic glasses^2–5^, the deformation of ferromagnetic shape-memory alloys^6^, nanoindentation^7^, the nucleation of one phase (crystal/droplet) in another phase (solution/gas)^8,9^ and the nucleation-controlled plasticity of crystalline solids^10,11^. The energy barrier can be changed by an external force and can even be eliminated, a case in which nucleation is spontaneous and no longer probabilistic. Thus, to control the strength of pristine specimen at the nanoscale, estimating the activation parameters for dislocation nucleation under different external driving forces, from which the nucleation rates can be estimated, is imperative.

The free-energy barrier is characterized by several parameters: the activation energy, volume and entropy. Calculating the activation parameters experimentally for dislocation nucleation remains quite challenging. Almost all experiments are performed by nanoindenting specimens at various temperatures, from which the first strain burst (known also as pop-in) is identified as dislocation nucleation^7,12,13^. The statistics over the first pop-in event is employed to calculate the activation parameters for dislocation nucleation beneath the indent. However, in the analysis of the nanoindentation experiments, it is postulated that the energy barrier decreases linearly with the stress, without any physical justification. Recently, a similar objective was addressed by Gianola and co-authors, who performed tensile experiments of heated Pd nanowires^11^. Despite the different experimental setup, the activation parameters in these experiments are also evaluated indirectly from the distribution of the measured strength. For this, the activation energy is assumed to have a power-law dependence on the stress with a postulated exponent equal either to 1 or to 4. The latter value was embraced from atomistic simulations^10^.

Indeed, dislocation nucleation is described naturally in atomistic simulations and direct calculation of the activation parameters for dislocation nucleation has been addressed computationally in recent years. As opposed to the experimental analysis, the activation energies were not extracted from simulations based on the probabilistic behavior of nucleation. Instead, minimum-energy path techniques are employed to calculate the activation energy^10,14–16^. For instance, Aubry *et al*. performed Nudged Elastic Band (NEB) calculations of the energy barrier to nucleate a dislocation in bulk Cu^17^. Zhu *et al*. performed free-end NEB calculations, which is a variation of the NEB method to explore strongly driven reactions, to study the dislocation nucleation in Cu nanowires under uniaxial compression^10^. A power-law was fitted to the function of the activation energy on the compressive stress, and an exponent of 4.1 was calculated. Although this value is large and was not observed in other nucleation mechanisms, it was left unexplained. It was even pointed out that the expected value of 1.5 might not apply to dislocation nucleation^14^. A similar simulation method was applied to Au nanowires of different shapes, albeit phenomenological functions were fitted to the activation energy function of the stress^15^.

While minimum energy techniques were employed to study the activation energies and volumes, the activation entropy, which corresponds to the nucleation rate, was computed using accelerated dynamic techniques^16,18^. Recently, a non-dynamic method was proposed to calculate the nucleation rates of dislocations, using umbrella sampling with forward-flux sampling^19,20^. The method, that was implemented both for homogeneous nucleation and for heterogeneous nucleation from the edges of Cu nanowires under compression, found significant activation entropies between a few ${k}_{B}$ and a few tens of ${k}_{B}$ (${k}_{B}$ is Boltzmann constant). It was pointed out that for these values the nucleation rate pre-factor is substantially higher than the Debye frequency.

The results above demonstrate the complexity of calculating the activation parameters for dislocation nucleation. Despite the well-established NEB simulation technique, the stress dependence of the activation energy is still left unresolved. In addition, a method like the umbrella sampling is still complex and beyond the reach of many to study dislocation nucleation. In this work, we profit from both the computational and the experimental analysis techniques to calculate the activation parameters; on the one hand, we employ atomistic simulations to probe directly the nucleation process, and on the other hand, we extract the activation parameters indirectly by analyzing the distribution of mechanical properties in the simulations. By using a varying external driving force, we can dynamically change the free-energy barrier during molecular dynamics (MD) simulation, allowing the system of atoms to fluctuate sufficiently around equilibrium in order to sample the phase space and also to constantly decrease the free-energy barrier to stimulate nucleation in the typical times of MD simulations. As we shall show, this varying driving force technique allows us to calculate the activation parameters for dislocation nucleation in Mo nanoparticles and, in particular, to study the stress-dependency of the free-energy barrier.

## Calculating Directly the Activation Volume from the Distribution Functions

Before discussing the calculation of the activation parameters for dislocation nucleation, we would like first to make a general discussion on how the dislocation-nucleation controlled strength is distributed as a function of the activation parameters. We denote the free-energy barrier a system needs to overcome in order to nucleate a dislocation by $G(\sigma ,T)$. The free-energy barrier depends on the stress $\sigma$ and the temperature $T$. For the sake of this discussion, we assume like others a first-order contribution of the temperature to the activation free-energy; $G(\sigma ,T)=Q(\sigma )-S(\sigma )T$ where $Q(\sigma )$ is the (athermal) activation energy and $S(\sigma )$ is activation entropy. Additionally, a linear empirical relation $S(\sigma )=Q(\sigma )/{T}_{m}$, also known as the Meyer-Neldel compensation rule^21^, is assumed. ${T}_{m}$ is a characteristic temperature at which the free-energy barrier vanishes for all stresses. Under these assumptions, the free energy barrier is expressed as:

1 $$G(\sigma ,{\rm{T}})=Q(\sigma )(1-{\rm{T}}/{{\rm{T}}}_{m}).$$

The activation volume of the thermally activated process is defined as the sensitivity of the free-energy to changes in the stress, i.e., ${{\rm{\Omega }}}_{G}(\sigma ,{\rm{T}})=-\partial G/\partial \sigma$. The separation of variables in Eq. (1) allows us to define the stress-dependent part of the activation volume as ${\rm{\Omega }}(\sigma )=-dQ/d\sigma$, which is related to the activation volume through ${{\rm{\Omega }}}_{G}(\sigma ,{\rm{T}})={\rm{\Omega }}(\sigma )(1-{\rm{T}}/{{\rm{T}}}_{m})$.

Without thermal fluctuations ($T=0$), the thermally activated process becomes an instability problem, in which the critical stress ${\sigma }_{0}$ satisfies $Q({\sigma }_{0})=0$ at the bifurcation point. This critical value at $T=0$ differentiates between stresses above which the thermally activated process occurs spontaneously and which it never occurs. If $T\ne 0$, the thermally activated processes can occur under stresses lower than ${\sigma }_{0}$, although $Q(\sigma ) > 0$. Thermal fluctuations allow the system to fluctuate in phase space around its metastable state and to overcome the energy barrier at a certain site. The rate at which the process occurs $\nu (\sigma ,{\rm T})$ satisfies

2 $$\nu (\sigma ,{\rm T})={\nu }_{0}^{0}{e}^{-\beta G(\sigma ,{\rm T})},$$

where ${{\rm{\nu }}}_{0}^{0}$ is a rate prefactor, and $\beta ={({k}_{B}T)}^{-1}$ has its usual meaning. While stress and temperature affect the rate mostly via the free energy, there are different models for the rate prefactor. For instance, based on harmonic transition state theory, the rate prefactor is stress- and temperature- independent. On the other hand, based on the Becker-Döring theory, the rate prefactor has two contributions from the molecular attachment rate and the Zeldovich factor. While both are not constant, Ryu *et al*. found ${{\rm{\nu }}}_{0}^{0}$ to slowly change as a function of stress and temperature^19^. Therefore, we shall assume here that ${\nu }_{0}^{0}$ is stress- and temperature- independent.

Considering the activation free-energy function in Eq. (1), the nucleation rate can be written as

3 $$\nu (\sigma ,{\rm{T}})={\nu }_{0}^{0}{e}^{-{\beta }^{\ast }Q(\sigma )},$$

where $${\beta }^{\ast }={({k}_{B}{T}_{eff})}^{-1}$$ and

4 $$\frac{1}{{T}_{eff}}=\frac{1}{T}-\frac{1}{{T}_{m}}.$$

Suppose a varying stress is applied to a system, increased at a constant rate $\sigma =\dot{\sigma }t$ at a given constant temperature $T$. As a result, owing to the probabilistic nature of thermally activated processes, the system will overcome the barrier at different stress levels, and the probability for the transition to occur before the stress reaches a certain level $\sigma$ is

5 $$F(\sigma ,T)=1-\exp [-\frac{{\rm{{\rm N}}}{{\rm{\nu }}}_{0}^{0}}{\dot{\sigma }}{\int }_{0}^{\sigma }{e}^{-{\beta }^{\ast }Q(\eta )}d\eta ].$$

$F(\sigma ,T)$ is also known as the cumulative distribution function (CDF). In the derivation of Eq. (5), it was assumed that there are only *N* possible nucleation sites of equal probability and that the system overcomes the barrier without recurring crossings. Given that the function $Q(\sigma )$ is known, the most probable stress for a dislocation to be nucleated $\bar{\sigma }$ can be obtained from the CDF, and it satisfies

6 $$\frac{{\rm N}{{\rm{\nu }}}_{0}^{0}}{\dot{\sigma }}{e}^{-{\beta }^{\ast }Q(\overline{\sigma })}={\beta }^{\ast }{\rm{\Omega }}(\overline{\sigma }).$$

The reader is referred to Supplementary Note 1 for more details on the derivation of the model. In the Supplementary Note, we obtained a relation for a general free energy function, using $\beta G(\overline{\sigma },{\rm{T}})$ and $\beta {{\rm{\Omega }}}_{G}(\overline{\sigma },{\rm{T}})$, without assuming the Meyer-Neldel compensation rule,

7 $$\frac{{{\rm{{\rm N}}}{\rm{\nu }}}_{0}^{0}}{\dot{\sigma }}{e}^{-\beta G(\overline{\sigma },{\rm{T}})}=\beta {{\rm{\Omega }}}_{G}(\overline{\sigma },{\rm{T}}).$$

When $T=0$, the CDF is the Heaviside step function $H(\sigma -{\sigma }_{0})$, and $\bar{\sigma }={\sigma }_{0}$ is the critical stress for a spontaneous nucleation. At finite temperatures, the CDF is a continuous function that varies from nearly zero to nearly one for stresses substantially below and above $\bar{\sigma }$, respectively. The super-exponential term in Eq. (5) leads to relatively narrow distributions, which allow CDF simplification by using a Taylor series expansion around $\bar{\sigma }$ up to the second order. As a result, the CDF takes the form of a normal distribution

8 $$F(\sigma ,T)=\frac{1}{2}[1+erf(\frac{\sigma -\overline{\sigma }}{\sqrt{2}\omega })],$$

where $\bar{\sigma }$ is found from Eq. (6), and the distribution width satisfies

9 $$\omega (\overline{\sigma },T)={[{({\beta }^{\ast }{\rm{\Omega }}(\overline{\sigma }))}^{2}-{\beta }^{\ast }{\rm{\Omega }}\text{'}(\overline{\sigma })]}^{-1/2}.$$

For the problem presented here, the second term beneath the square root in Eq. (10) is negligible with respect to the first term. Consequently, the activation volume can be calculated directly from the standard deviation of the distribution function

10 $${\rm{\Omega }}(\overline{\sigma })\approx \frac{{k}_{B}{T}_{eff}}{\omega (\overline{\sigma },T)},$$

or alternatively, without assuming the Meyer-Neldel compensation rule,

11 $${{\rm{\Omega }}}_{G}(\bar{\sigma },T)\approx \frac{{k}_{B}T}{\omega (\bar{\sigma },T)}.$$

This is a key finding of the model presented here. If one could produce, either computationally or experimentally, the CDF at a given temperature while varying the driving force at a constant rate, the activation volume can be extracted directly from the distribution of the results. If we recall the definition of the activation volume, i.e., the sensitivity of the free-energy barrier to changes in the driving force, a wider distribution of the CDF means that the free-energy barrier changes less with an increase in the driving force, i.e., the activation volume is smaller. Knowing the dependency of the activation volume on the stress and temperature will enable quantifying the activation energy as a function of the stress and temperature, from which the dislocation nucleation rate and the activation entropy can be calculated. This principle is demonstrated here on the calculation of the activation parameters for dislocation nucleation in Mo nanoparticles.

## Activation parameters for dislocation nucleation in Mo nanoparticles

We apply the model to the problem of heterogeneous dislocation nucleation and we study the activation parameters for dislocation nucleation in Mo nanoparticles under compression using classical molecular dynamics (MD) simulations. We employ for that the Large-scale Atomic/Molecular Massively Parallel Simulator (LAMMPS)^22^. Since the approach proposed here requires increasing the stress at a constant rate until a nucleation event occurs, it is naturally applied to MD simulations, and the activation parameters for dislocation nucleation near the conditions of spontaneous nucleation can be explored in the timescales of MD simulations. We refer the reader to the Methods section for further details on the MD simulations.

**Figure 1:**
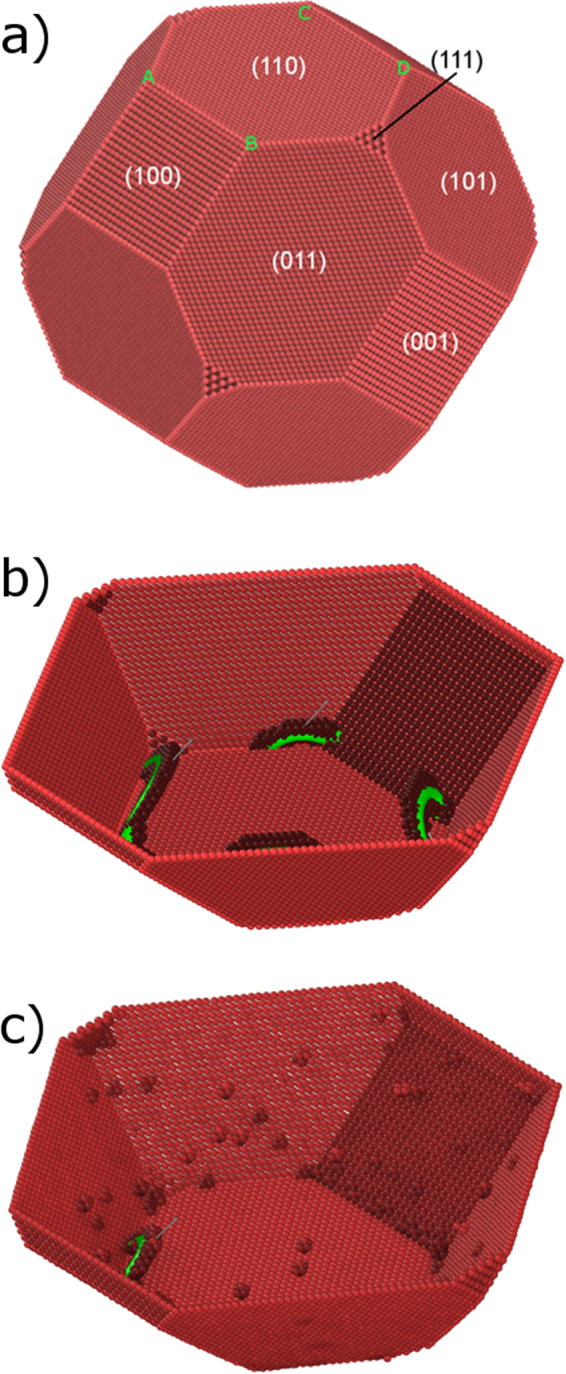
Dislocation nucleation in a 19.8-nm-high Mo nanoparticle. (**a**) The shape of the faceted nanoparticle. The Miller indices of several representative facets are noted. The vertices on which dislocations were found to nucleate are marked by A, B, C and D. (**b**) The onset of plasticity at 10 K. Four dislocations were found to nucleate on the vertices of the compressed facet. For simplicity, only one side of the particle is shown, and the atoms that are in perfect BCC positions are not shown. The dislocation lines and Burgers vector are also plotted. (**c**) Similar to (**b**) but at 800 K.

Based on the 0 K surface energies, the nanoparticles exhibit a faceted shape, with facets on the $\{110\}$, $\{100\}$ and {111} planes, as shown in Fig. 1a. Under compression along the $[110]$ direction, dislocations are nucleated at four out of the eight vertices of the compressed {110} facet, i.e., there are $N=8$ most-probable nucleation sites in total. When the atomic velocities are initialized to 10 K (thermal energy is negligible), nucleation occurs almost spontaneously. As with Fe nanoparticles^23^, all dislocations are nucleated in the ${1/2}\langle 111\rangle \{110\}$ slip system (Fig. 1b). The potential nucleation sites and slip systems did not vary at higher temperatures (e.g., Fig. 1c at 800 K). Accordingly, we conclude that the probability of nucleating dislocations these eight vertices is substantially higher than at other sites along the facets, within the range of temperatures examined here.

**Figure 2:**
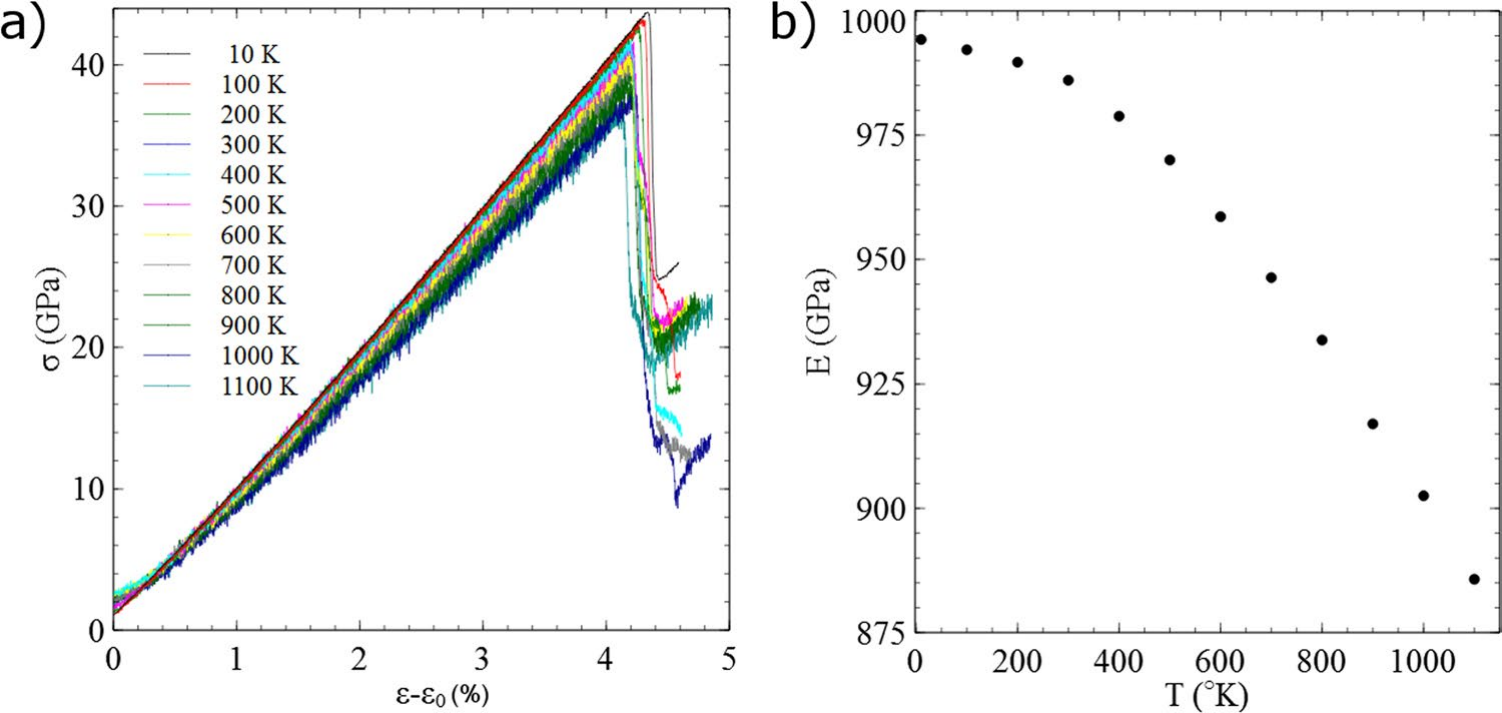
The temperature-dependent elastic response of a 19.8-nm-high Mo nanoparticle. (**a**) Stress-strain curves at different temperatures. Since the slope is not linear in the early stages of the deformation, the strain is shifted by ε_0_, so that all curves start approximately at the same stress at the onset of the linear elastic regime. The shift allows a visible comparison of the slopes. (**b**) The effective Young’s modulus as a function of temperature.

The calculation of the activation parameters is demonstrated on a 19.8-nm-high nanoparticle compressed at a constant strain rate. The compressive stress σ is recorded during the deformation. Several representative stress-strain curves at different temperatures are shown in Fig. 2a. At very small strains the deformation is nonlinear. Nonlinear elasticity in pristine nano-specimens was identified both experimentally^24^ and computationally^25^, which was attributed to surface stress effects. The nonlinear stress-strain response at the early stages may be a result of the large surface-to-volume ratio in the nanoparticles but also due to the errors in calculating the contact area due to the roundness of the relaxed surface^26^. Once the facets reach full contact with the indenters, a clear linear elastic regime is identified in all temperatures, terminated by an abrupt drop in the load. Hence, the compressive stress rate is constant and related to the strain through $\dot{\sigma }=E\dot{\varepsilon }$, where $E(T)$ is an effective Young’s modulus and $\dot{\varepsilon }$ is the engineering strain rate. The values of $E(T)$ were calculated directly from the slopes of the stress-strain curves in the linear elastic regime, and the results as a function of the temperature are shown in Fig. 2b.

**Figure 3:**
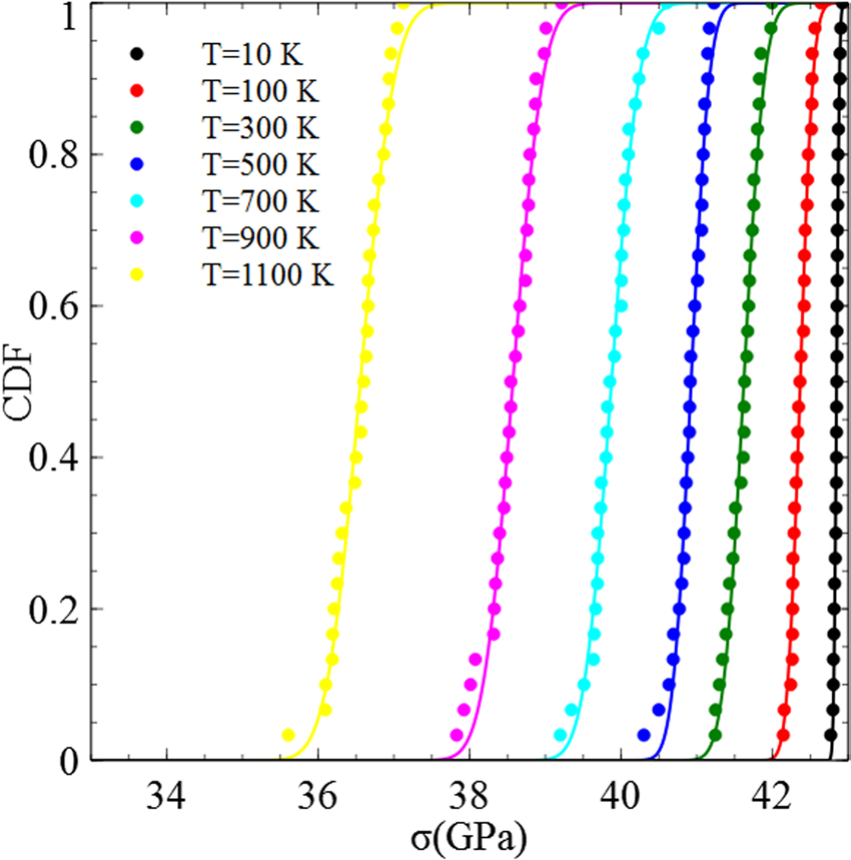
CDFs of the compressive stresses at nucleation for various temperatures.

**Figure 4:**
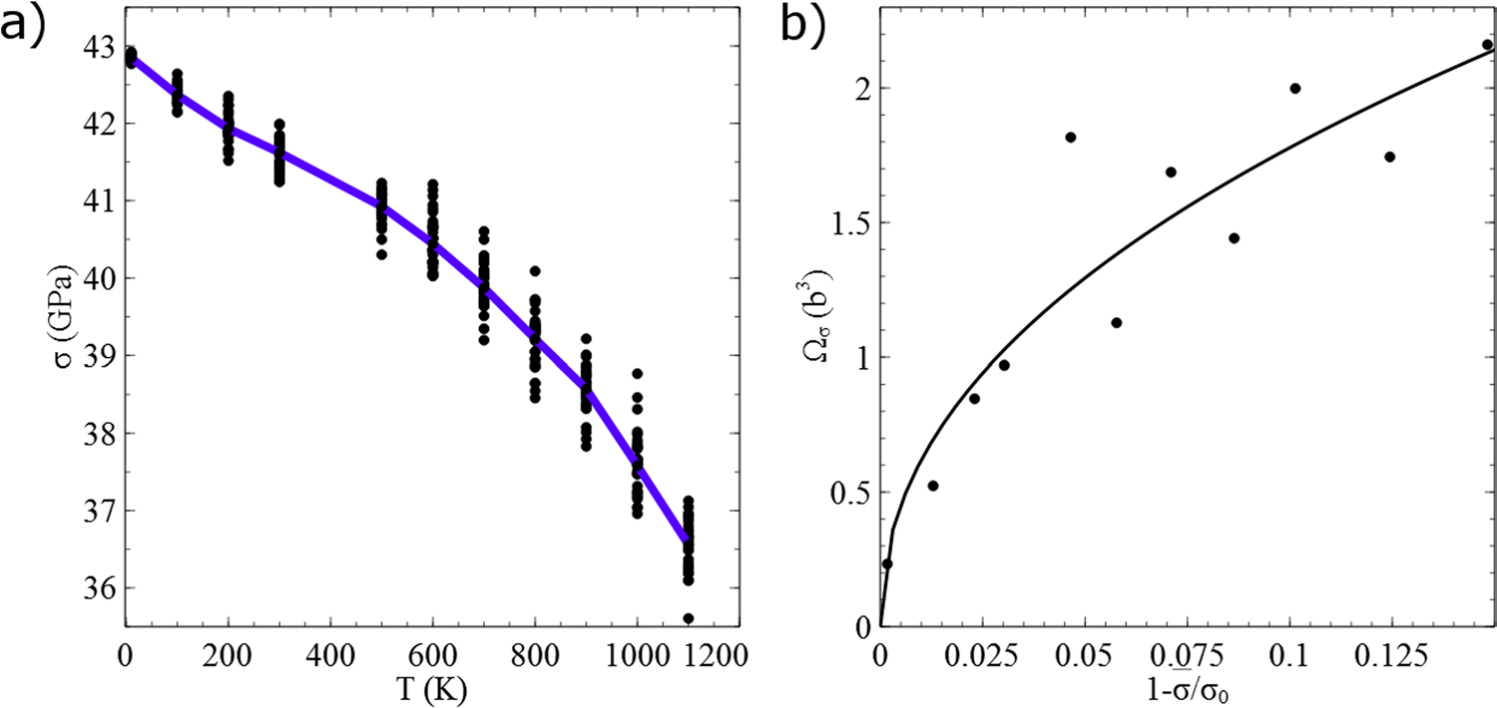
MD simulation results of dislocation nucleation at the vertices of a 19.8-nm-high Mo nanoparticle. (**a**) The mean compressive stress for nucleation at various temperatures (blue line). The 30 values of the compressive stress at the onset of plasticity at each temperature are summarized in black dots. (**b**) The activation volume as a function of $1-\overline{\sigma }/{\sigma }_{0}$. A power-law fit is plotted, made under the assumption that ${T}_{m}\gg T$.

The load drop is associated with dislocation nucleation. For each temperature, the simulation is repeated 30 times. In each set of simulations, the compressive stresses at the onset of plasticity were calculated, and a CDF of the compressive stresses at nucleation was constructed. In Fig. 3, we plot a few representative CDFs at different temperatures. As expected from a thermally activated process, at low temperatures the CDF approached a Heaviside step function and as the temperature is increased, nucleation does not occur at the same compressive stress but being distributed between the 30 simulations. Fitting normal distributions [Eq. (8)] to the results yields the mean compressive stresses for nucleation $\bar{\sigma }$ at different temperatures. The mean compressive stress for dislocation nucleation decreases with the temperature (Fig. 4a). Extrapolating the curve to 0 K, we estimate the compressive stress for spontaneous nucleation to be ${\sigma }_{0}$ = 42.9 GPa.

The standard deviation of the normal distribution yields the activation volume at different temperatures and stresses based on Eq. (11). In Fig. 4b, we plot the activation volume ${{\rm{\Omega }}}_{G}^{(\sigma )}$ as a function of $1-\overline{\sigma }/{\sigma }_{0}$. The superscript indicates that the activation volume refers to the sensitivity of free-energy barrier to the compressive stress and not to the resolved shear stress (RSS) $\tau$. In fact, the driving force that controls the activation energy in this case is the RSS on the slip planes on which the dislocations nucleate. However, we employ σ since it is a more accessible parameter in the simulations. We show in Supplementary Note 2 that while the activation energy and activation entropy, calculated based on the compressive stress, are equal to those calculated using the RSS, the activation volume that corresponds to the RSS ${{\rm{\Omega }}}_{G}^{(\tau )}$ is equal to ${{\rm{\Omega }}}_{G}^{(\sigma )}$, multiplied by the ratio between the compressive stress and the RSS at the nucleation site. This ratio is composed of the Schmid factor $m=0.433$ and the stress concentration. Since the latter is difficult to be determined exactly, one should consider the values in Fig. 4b, multiplied by ${m}^{-1}=2.31$, as an upper bound for ${{\rm{\Omega }}}_{G}^{(\tau )}$. Nonetheless, the values are within the range of a few ${b}^{3}$ ($b$ is the Burgers vectors), which are the characteristic values found in atomistic simulations using minimum-energy path techniques^10^.

Let us first neglect the entropic contribution (${T}_{m}\gg T$). In this case, we can assume that the activation volume is only a function of the stress ${{\rm{\Omega }}}_{G}(\sigma ,T)\approx {\rm{\Omega }}(\sigma )$. The activation volume increases as nucleation occurs at lower mean compressive stresses. More quantitatively, near the critical stress or spontaneous nucleation the energy barrier is customarily being considered to follow a power-law:

12 $$Q(\sigma )={Q}_{0}{(1-\frac{\sigma }{{\sigma }_{0}})}^{\alpha },$$

where ${Q}_{0}$ and $\alpha$ are calibration parameters. The best fit of the activation volumes to this relation is obtained with ${Q}_{0}=18.92eV$ and $\alpha =1.46$ (solid line in Fig. 4b). While the value of ${Q}_{0}$ was not calculated previously for Mo, free-end nudged elastic band simulations of Cu nanowires under compression yielded values of ${Q}_{0}=4.8eV$ and ${\sigma }_{0}=5.2GPa$. Although a different exponent α was found there, the critical stress found here is approximately 8 times larger than that found for Cu; hence, the value obtained here for ${Q}_{0}$ is reasonable. The value of the exponent $\alpha$, which is very close to a value of 1.5, appears in other different systems (when neglecting the contribution of the activation entropy). In some studies^7,12,13^, a constant activation volume is considered ($\alpha =1$), whereas in others^11^, both $\alpha =1$ and $\alpha =4$ were chosen. The latter value is based on atomistic simulations of Cu nanowires^10^, in which a value of $\alpha =4.1$ was proposed. These values have no theoretical basis and were mainly extracted from fitting atomistic simulation results. In fact, a value of $\alpha =1.5$ was previously proposed by Cahn and Nabarro^27^ and by Cottrell^28^, who argued that the general activation barriers near bifurcation-type event depend exponentially on the stress with this exponent. Until now, it was believed that this exponent is not applicable for the problem of dislocation nucleation since it was not identified in simulations^14^. However, we recall that spontaneous nucleation can be treated as an instability at a bifurcation point^29^ and the exponent of the energy barrier near the critical stress found here is in outstanding agreement with the theoretical predictions. We suggest that the reason we identified this exponent for the first time is due to the sampling of configuration space with MD simulations near the bifurcation point.

However, in the MD simulations we reach a temperature of 1100 K, which is of the order to the typical values for ${T}_{m}$ for surface nucleation. Thus, the activation volume at higher temperatures includes also non-negligible contributions of the temperature, through the activation entropy. In this case, the free energy barrier obeys

13 $$G(\sigma ,T)={Q}_{0}{(1-\frac{\sigma }{{\sigma }_{0}})}^{\alpha }(1-\frac{T}{{T}_{m}}).$$

Since the value of ${T}_{m}$ is unknown, the values ${Q}_{0}$ and $\alpha$ are calculated in a self-consistent method. First, a value for ${T}_{m}$ is chosen and the stress-dependent part of the activation volume ${\rm{\Omega }}(\sigma )$ is obtained by dividing ${{\rm{\Omega }}}_{G}^{(\sigma )}(\sigma ,T)$ by $1-T/{T}_{m}$. Fitting an activation energy of the form of Eq. (12) yields the values ${Q}_{0}$ and $\alpha$ as a function of ${T}_{m}$. Using the fitted expression for the activation free-energy barrier, the CDF in Eq. (5) is calculated explicitly, without the need to approximate the results to a normal distribution,

14 $$F(\sigma ,T)=1-\exp \langle -\frac{{{\rm{{\rm N}}}{\rm{\nu }}}_{0}^{0}{\sigma }_{0}}{E\dot{\varepsilon }\alpha {({\beta }^{\ast }{Q}_{0})}^{1/\alpha }}\{{\rm{\Gamma }}[\frac{1}{\alpha },{\beta }^{\ast }G(\sigma ,T)]-{\rm{\Gamma }}[\frac{1}{\alpha },{\beta }^{\ast }G(0,{\rm{T}})]\}\rangle ,$$

where ${\rm{\Gamma }}$ is the incomplete upper gamma function. The calculated CDFs are compared with the CDFs obtained from the MD simulations and the best fit is obtained for ${T}_{m}$ = 1650 K and ${{\rm{\nu }}}_{0}^{0}$ = 1.3·10^13 ^sec^−1^. Details of the analysis are provided in Supplementary Note 3. The corresponding ${Q}_{0}$ and $\alpha$ are 40.76 eV and 1.68, respectively. One can see that the exponent increased slightly, with respect to the value obtained without the activation entropy. However, the activation energy pre-factor was underestimated without including the activation entropy and a value as twice as large is obtained.

**Figure 5:**
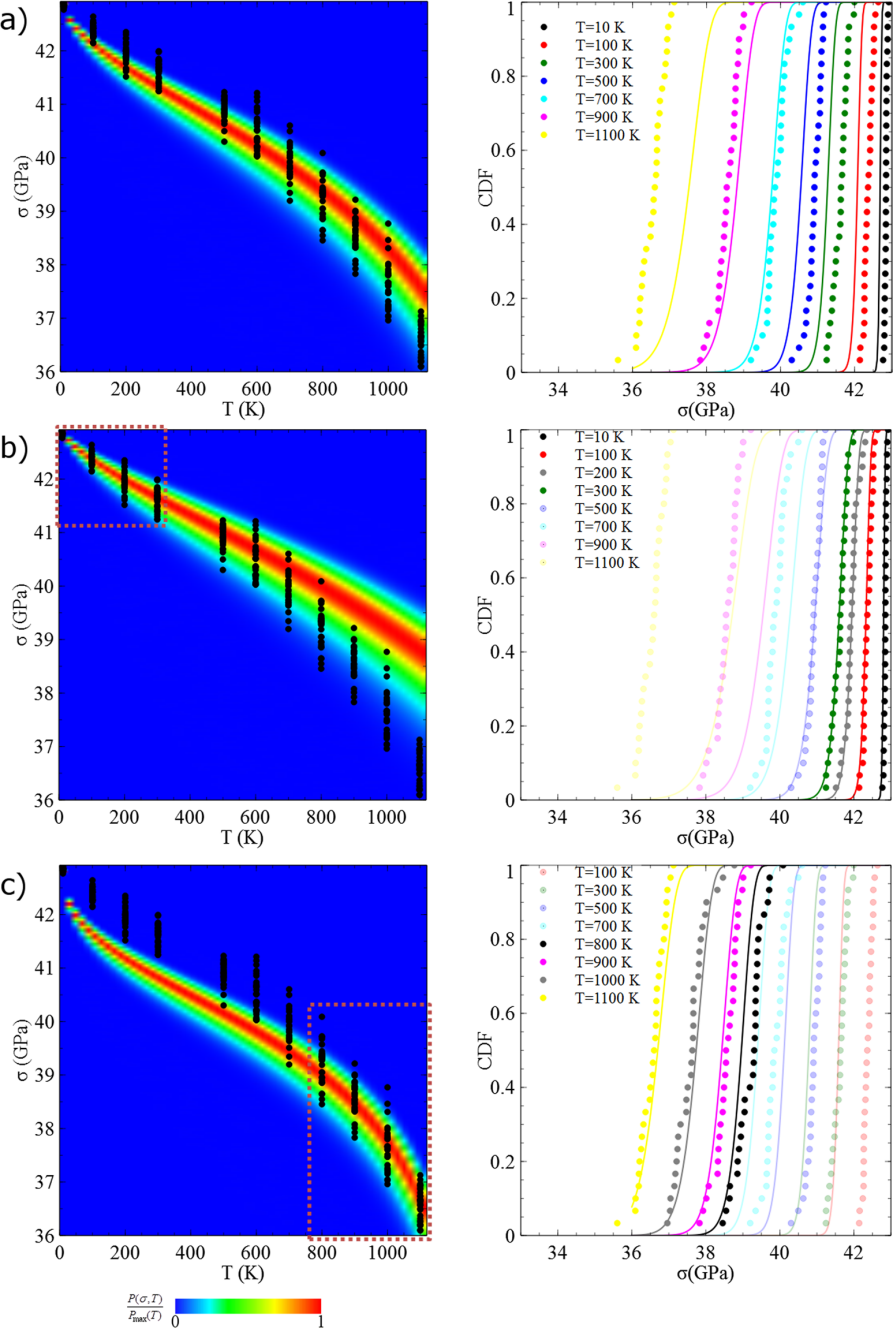
The PDFs and CDFs calculated with (**a**) ${T}_{m}$ = 1650 K and ${\nu }_{0}^{0}$ = 1.3·10^13^ sec^−1^, (**b**) ${T}_{m}$ = 2250 K and ${\nu }_{0}^{0}$ = 4·10^12^ sec^−1^ and (**c**) ${T}_{m}$ = 1300 K and ${\nu }_{0}^{0}$ = 1.3·10^13^ sec^−1^. The parameters in (a), (b) and (c) yield the best fit to all CDFs, to the four lowest temperatures and to the four highest ones, respectively. The color map in the nucleation stress-temperature curves indicate the value of the PDF at a certain stress and temperature $P(\sigma ,T)$, scaled by the maximum value at a given temperature ${P}_{\max }(T)$. The semi-transparent data points in the CDFs in (b) and (c) are not included in the fit and the semi-transparent curves are calculated based on the fit to the other data points (marked also in dashed square in the PDF plots).

The theoretical CDF and probability density function (PDF), which is the stress-derivative of the CDF in Eq. (14), with the fitted values, are shown in Fig. 5a. The best fit obtained is in good agreement with the results, but the calculated most-probable nucleation stress is slightly underestimated at low temperatures and overestimated at higher temperatures. It is worth pointing out that the best fit minimizes the difference between the theoretical CDF and the MD-obtained one, but the difference does not change dramatically for different values of ${T}_{m}$ above approximately 1500 K (see Fig. S2 in the Supplementary Document). This suggests that different values of ${T}_{m}$ may correspond to different stress regimes. For this, the fit was limited to the four lowest temperatures (Fig. 5b) and the four highest temperatures (Fig. 5c). Fitting the CDFs to MD results at $T\le 300\,{\rm{K}}$ or at $T\ge 800\,{\rm{K}}$ leads to ${T}_{m}$ = 2550 K and ${{\rm{\nu }}}_{0}^{0}$ = 4·10^12^ sec^−1^ or ${T}_{m}$ = 1300 K and ${{\rm{\nu }}}_{0}^{0}$ = 1.3·10^13^ sec^−1^, respectively. The agreement between the CDFs in the fitted regions is substantially better, but the over- and underestimations in nucleation stresses in the non-fitted region increases. Nonetheless, the stress- and temperature-independent rate prefactor is of the order of the Debye frequency, and changes only slightly for different stress and temperature regimes, as was also found using the Becker-Döring theory for heterogeneous nucleation of dislocations in Cu nanorods (${{\rm{\nu }}}_{0}^{0}$ = 5·10^12^ sec^−1^ on average)^19^.

Considering the values fitted to the whole set of MD results, the activation energy varies between 0 and 2.13 eV in the range of stresses calculated in the MD simulation (35.5–42.9 GPa). Given the fitted value for ${T}_{m}$, the activation entropies are found to be between 0 and 15 ${k}_{B}$ eV/K. These values demonstrate the immense contribution of the activation entropies to the nucleation rates. The computed values are of the same magnitudes as those obtained by Ryu *et al*. for the homogeneous and heterogeneous nucleation of dislocations in Cu nanorods in the constant-stress ensmble^19,20^. Using a different computational method (activation-relaxation technique *nouveau*), similar activation entropies for heterogeneous nucleation of dislocations in Cu nanorods of different cross-sections were found^30^.

The fitted value of ${T}_{m}$ in our work is 54% of the bulk melting temperature (found to be 3062 K in ref.^31^). Other studies found similar values, between 61–68% of the bulk melting temperature in heterogeneous nucleation in Cu^19,20,30^ and between 74–81% of the bulk melting temperature for creep of nanopolycrytalline Cu^32^. Some suggest that ${T}_{m}$ corresponds to the melting temperature on the surface. Based on the Lindemann criterion, it was proposed that the surface melting temperature is approximately 66% of the bulk value since surface atoms have a smaller coordination than bulk atoms^33^. At the vertices of the nanoparticles, where the dislocations are nucleated, the coordination number is even lower, which may even reduce the melting temperature at the nucleation sites. However, the relation between ${T}_{m}$ and the melting temperature at the nucleation sites is still a matter of debate. For instance, the average ${T}_{m}$ found in heterogonous dislocation nucleation in constant-strain ensemble was found to be 180% of the bulk melting temperature. Examining the relation between the fitted value of ${T}_{m}$ and the melting temperature at the vertices of the Mo nanoparticles is planned in the future, where the latter value will be quantified.

The assumption that ${T}_{m}$ is constant in the whole range of temperatures is based on the postulated Meyer-Neldel compensation rule. Nonetheless, although the compensation rule is commonly believed not to be violated, it is not always the case (e.g. for heterogeneous dislocation nucleation in Cu nanorods in constant-strain ensemble). We suggest that the different values of ${T}_{m}$ fitted for the lowest and highest temperatures is indicative of a non-linear relation between the activation entropies and energies, in the range of activation energies calculated from the MD simulations; the slope of the activation energy-entropy relation is smaller at lower temperatures (lower activation energies) than at higher temperatures, similarly to the results in Cu nanorods in constant-strain ensemble^19^. While extension of the model presented here to non-linear activation entropy-energy relations is beyond the scope of this work, we emphasize that Eq. (7) (for the temperature dependence of the most probable nucleation stress) and Eq. (11) (relating the nucleation stress distribution and the activation volume) are independent of the relation between the activation entropy and the energy.

## Conclusions

In this work, we proposed a method to calculate indirectly the activation parameters for dislocation nucleation in Mo nanoparticles using MD simulations. Although MD simulations are limited in their simulated time, constantly increasing the driving force (compressive stress) allows exploring configurational space near spontaneous nucleation. As a result, our analysis showed for the first time, to the best of our knowledge, that the critical exponent of the activation barrier near the spontaneous nucleation threshold is ~1.5, as expected from a simple bifurcation problem^29^. Although this exponent was not calculated before for dislocation nucleation, a similar exponent was calculated for shear transformation in glasses^5^ and was proposed to be also true for molecular systems^34^. It is worth mentioning that the exponent of 1.5 was proposed theoretically near the spontaneous nucleation threshold, and for this reason it is probably captured within the timeframe of the MD simulation. The higher exponent found in ref.^10^ was obtained using minimum-energy techniques, which allows exploring the energy barrier far from the bifurcation point, in regimes that are not accessible for ordinary MD simulations. Additionally, nucleation in experimental conditions can be estimated using Eq. (7), with the experimental strain-rate, bearing in mind that if nucleation occurs far from the spontaneous nucleation conditions, larger exponents may also be required. Nonetheless, the model presented here can be applied on experimental results, in order to calculate the activation parameters in the stress and temperature regimes explored experimentally. The calculated activation entropies are also in good comparison with the values obtained using the umbrella sampling, despite the time limitation of the MD simulations.

The approach adopted here is not limited to dislocation nucleation but can be generalized for driving-force dependent thermally activated problems. In Supplementary Note 1, we formulated the model for a general driving force λ. Such a driving force can be mechanical, electric or magnetic, e.g. the motion of twin wall ferromagnetic shape memory alloys under a magnetic driving force^35^. Varying the driving force at a constant rate allows the direct extraction of the activation energies from the CDFs and the model can be implemented to nucleation of droplets, phase transformation, shape-memory properties, dislocation cross-slip and more. Such an understanding is imperative for the microstructural design of thermally activated nanoscale systems and for predicting their probabilistic behavior at different temperatures.

## Methods

### Molecular Dynamics Simulations

The MD simulations were performed using LAMMPS^22^. The interatomic interactions were described according to the embedded atom method, with parameterizations for Mo^36^. The initial shape of the nanoparticle was determined using the Wulff construction, which minimizes the total surface energy of the nanoparticle. For the construction, we calculated the anisotropic relaxed surface energies obtained with the potential at 0 K: ${\gamma }_{111}=0.228$ eV Å^−2^, ${\gamma }_{110}=0.192$ eV Å^−2^ and ${\gamma }_{100}=0.203$ eV Å^−2^.

The nanoparticle was initially statically relaxed via a conjugate gradient, followed by a dynamic simulation in the canonical ensemble, controlling the temperature using a Langevin thermostat. The time step during the dynamic stage was set to 3 fsec. Before beginning the compression, the nanoparticle was relaxed dynamically over 10,000 MD time steps. Compression was then accomplished by moving two virtual, flat indenters, which were parallel to two opposite {110} facets, perpendicularly to the facets. The force was proportional to the square of the distance from the indenter, with a proportion constant equal to 10 eV Å^−3^. Each virtual indenter was subjected to a constant displacement of 0.005 Å ps^−1^ toward one another. The engineering stress was defined as the force acting on each indenter divided by the undeformed and relaxed contact area between the indenter and the facet. The engineering strain was defined as the relative change in the height of the nanoparticle. Defects were identified with the common neighbors analysis and the dislocation extraction algorithm, and atoms were visualized using the visualization tool Ovito^37^.

## Electronic supplementary material

Supplementary Information
