# Supplementary material for: The Stress-Dependent Activation Parameters for Dislocation Nucleation in Molybdenum Nanoparticles
Source: Sci Rep. 2018 Mar 2;8:3915. doi: 10.1038/s41598-018-21868-y (PMC5834640; doi:10.1038/s41598-018-21868-y)
Supplement: Supplementary file 1 — Supplementary Information [file 41598_2018_21868_MOESM1_ESM.pdf]

# Supplementary Information:

## The Stress-Dependent Activation Parameters for Dislocation Nucleation in Molybdenum Nanoparticles

Doron Chachamovitz and Dan Mordehai

Mechanical Engineering, Technion, 32000, Israel

Notation remark: In this document we notate partial derivatives with subscripts after a comma, e.g. the $\partial^2 f(x,y)/\partial x\partial y = f_{,xy}$.

**Note 1: Calculating the Activation Volume Directly from Distribution Functions**

Consider a thermally activated process with an activation free-energy $G(\lambda,\mathrm{T})$ where T is the temperature of the system and λ is the driving force. In the problem tackled in this work, λ is the compressive stress σ. Based on models, such as the transition rate theory or Becker-Döring theory, the rate at which the system overcomes this energy barrier is

(S1) $$\nu(\lambda,\mathrm{T}) = \nu_0^0 e^{-\beta G(\lambda,\mathrm{T})},$$

where $\nu_0^0$ is a stress-independent rate prefactor and $\beta = (k_B T)^{-1}$ has its usual meaning. In the method proposed here, we increase the driving force from zero at a constant rate $\lambda = \dot{\lambda} t$. The system will then fluctuate around its equilibrium state until overcoming the barrier at a certain time. The probability $F(\mathrm{t}+\mathrm{dt},T)$ of having a single passage over the barrier until time $t+dt$ is the probability to overcome the barrier either until time $t$ or during $dt$,

(S2) $$F(t+dt,T) = F(t,T) + \left[1 - F(t,T)\right] \mathrm{N}\nu(\dot{\lambda} t,\mathrm{T})dt,$$

Here it is assumed that there are $N$ possible nucleation sites with the same rate. The solution of this differential equation yields the function $F$, which is also known as the cumulative distribution function (CDF),

(S3) $$F(\lambda,T) = 1 - \exp\left[-\frac{\mathrm{N}\nu_0^0}{\dot{\lambda}} \int_0^{\lambda} e^{-\beta G(\eta,\mathrm{T})} d\eta\right].$$

Owing to the one-to-one relation between the driving force and the time, we replaced variables in the CDF to $\lambda$ and $T$. Its first derivative $F_{,\lambda}(\lambda,T)$, known also as the

probability density function (PDF), is the probability that the transition would occur while rising the driving force from $\lambda$ by $d\lambda$,

(S4) $$F_{,\lambda}(\lambda,T)=\frac{\mathrm{N}\nu_0^0}{\dot{\lambda}}\exp\left[-\frac{\mathrm{N}\nu_0^0}{\dot{\lambda}}\int_0^{\lambda}e^{-\beta G(\eta,\mathrm{T})}d\eta-\beta G(\lambda,\mathrm{T})\right].$$

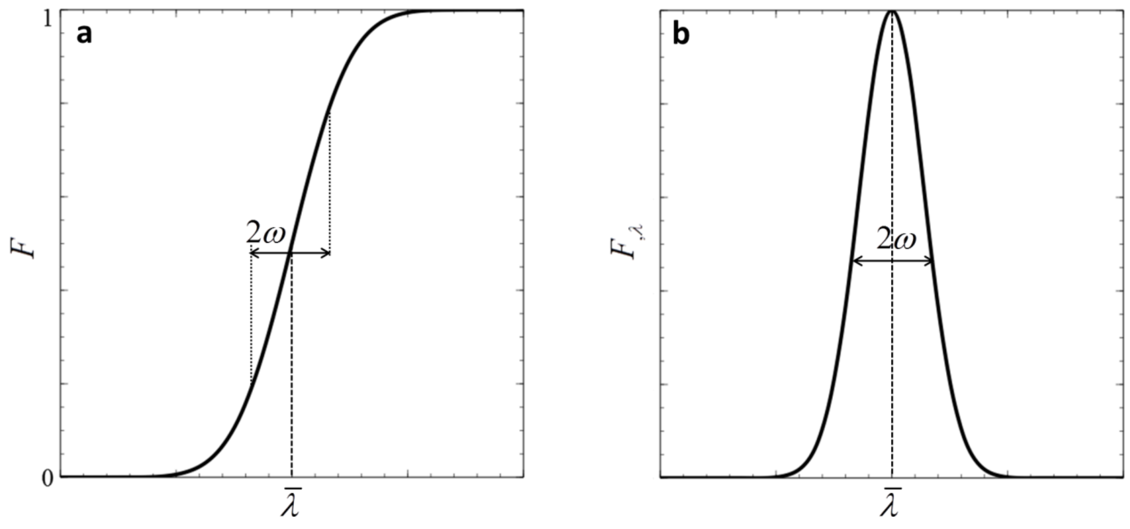


Figure S1: Schematics of the (a) CDF and (b) PDF.

In Fig. S1 the CDF and PDF are plotted schematically. $\bar{\lambda}$ is the driving force at which it is the most probable for the system to overcome the barrier and $\omega$ is a measure for the width of the distribution. The exact definition of this width will be further clarified. At the most probable driving force to overcome the barrier, the PDF is at maximum, i.e., the second stress derivate of the CDF is $F_{,\lambda\lambda}=0$. The second derivative with respect to the driving force of the CDF in Eq. (S3), is

(S5) $$F_{,\lambda\lambda}(\lambda,T)=\frac{\mathrm{N}\nu_0^0}{\dot{\lambda}}\exp\left\{-\frac{\mathrm{N}\nu_0^0}{\dot{\lambda}}\int_0^{\lambda}e^{-\beta G(\eta,T)}d\eta-\beta G(\lambda,T)\right\}\left\{-\frac{\mathrm{N}\nu_0^0}{\dot{\lambda}}e^{-\beta G(\lambda,T)}-\beta G_{,\lambda}(\lambda,T)\right\}.$$

The demand that $F_{,\lambda\lambda}(\bar{\lambda},T)=0$ yields the relation

(S6) $$\frac{\mathrm{N}\nu_0^0}{\dot{\lambda}}e^{-\beta G(\bar{\lambda},T)}=\beta\Omega_G(\bar{\lambda},\mathrm{T})$$

where the activation volume is defined as $\Omega_G(\lambda,T)=-G_{,\lambda}(\lambda,T)$. We note that the activation volume may obtain different values for the same driving force, but at different temperatures.

Fitting Eq. (S3) to experimental/computational results brings with it several challenges. First of all, *a-priori* explicit expression of the function $G(\lambda,\mathrm{T})$ is needed. For instance, in the work of Gianola and co-authors[1], equation of a similar form to

that of Eq. (S3) was fitted to experimental results of dislocation nucleation during tensile experiments of Pd nanowires at room temperature. The function $G(\lambda,\mathrm{T})$ was expressed explicitly as a power-law as a function of the driving force, with a postulated exponent of 4, and a linear relation with temperature, with a postulated temperature $T_m$ at which $G(\lambda,\mathrm{T}_m)=0$, in order to fit the other parameters to experimental results. In addition, we note that Eq. (S3) is strongly varying with the driving force (it is being exponentiated twice). As a result, fitting activation parameters from a set of experimental/simulation results leads to very large uncertainties. Therefore, we wish to simplify the expression, exploiting the fact that we expect the CDF and PDF to behave schematically as drawn in Fig. S1.

Let us define the function

(S7) $$H(\lambda,T)=-\frac{\mathrm{N}\nu_0^0}{\dot{\lambda}}\int_0^{\lambda} e^{-\beta G(\eta,T)}d\eta-\beta G(\lambda,T).$$

This function can be expanded in a Taylor series around $\bar{\lambda}$, neglecting terms of third order and above,

(S8) $$H(\lambda,T)\approx H(\bar{\lambda},\mathrm{T})+\left(-\frac{\mathrm{N}\nu_0^0}{\dot{\lambda}}e^{-\beta G(\bar{\lambda},\mathrm{T})}+\beta\Omega_G(\bar{\lambda},\mathrm{T})\right)(\lambda-\bar{\lambda})$$
$$+\frac{1}{2}\left(-\frac{\mathrm{N}\nu_0^0}{\dot{\lambda}}\beta\Omega_G(\bar{\lambda},\mathrm{T})e^{-\beta G(\bar{\lambda},\mathrm{T})}+\beta\Omega_{\mathrm{G},\lambda}(\bar{\lambda},\mathrm{T})\right)(\lambda-\bar{\lambda})^2.$$

According to Eq. (S6), the linear term nullifies. In addition, using Eq. (S6) in the second order term, results in the following expression

(S9) $$H(\lambda,T)\approx H(\bar{\lambda},\mathrm{T})+\frac{1}{2}\left[-\left(\beta\Omega_G(\bar{\lambda},\mathrm{T})\right)^2+\beta\Omega_{\mathrm{G},\lambda}(\bar{\lambda},\mathrm{T})\right](\lambda-\bar{\lambda})^2.$$

Finally, we note that $\mathrm{H}(\lambda,T)$ is the function that appears in the exponent of the PDF in Eq. (S4). Substituting the approximated function for $\mathrm{H}(\lambda,T)$ in the PDF leads to the following distribution function

(S10) $$F_{,\lambda}(\lambda,T)\approx\frac{\mathrm{N}\nu_0^0}{\dot{\lambda}}e^{H(\bar{\lambda},\mathrm{T})}e^{-\frac{(\lambda-\bar{\lambda})^2}{2\omega^2}},$$

where

(S11) $$\omega=\left(\left[\beta\Omega_G(\bar{\lambda},T)\right]^2-\beta\Omega_{\mathrm{G},\lambda}(\bar{\lambda},T)\right)^{-1/2}.$$

Eq. (S10) is a characteristic equation of a normal distribution centered at $\bar{\lambda}$, with a standard deviation $\omega$. Consequently, the CDF is approximately equal to

(S12) $$F(\lambda,T)\approx\frac{1}{2}\left[1+erf\left(\frac{\lambda-\bar{\lambda}}{\sqrt{2}\omega}\right)\right].$$

If $\left[\beta\Omega_G(\bar{\lambda},\mathrm{T})\right]^2\gg\beta\Omega_{G,\lambda}(\bar{\lambda},\mathrm{T})$, Eq. (S11) can be simplified

(S13) $$\omega(\bar{\lambda},\mathrm{T})\approx\frac{1}{\beta\Omega_G(\bar{\lambda},\mathrm{T})}.$$

While the model above is derived for a general free-energy barrier, in many cases it is assumed that the free energy barrier is linear with the temperature, i.e. $G(\lambda,\mathrm{T})=\mathrm{Q}(\lambda)-\mathrm{S}(\lambda)\mathrm{T}$ where $\mathrm{Q}(\lambda)$ and $\mathrm{S}(\lambda)$ are the activation energy and activation entropy, respectively. In addition, the compensation rule is usually postulated $S(\lambda)=Q(\lambda)/\mathrm{T}_m$ (we emphasize that the condition $G(\lambda,\mathrm{T}_m)=0$ is fulfilled). The contributions of the driving force and temperature to the free-energy barrier can then be treated separately

(S14) $$G(\lambda,\mathrm{T})=Q(\lambda)(1-\mathrm{T}/\mathrm{T}_m),$$

and the activation volume can be written as

(S15) $$\Omega_G(\lambda,\mathrm{T})=\Omega(\lambda)(1-\mathrm{T}/\mathrm{T}_m),$$

where $\Omega(\lambda)=-dQ(\lambda)/\mathrm{d}\lambda$ is the stress-dependent part of the activation volume. Under these assumptions, Eqs. (S6) and (S13) can be written as

(S16) $$\frac{\mathrm{N}\nu_0^0}{\dot{\lambda}}e^{-\beta^*Q(\bar{\lambda})}=\beta^*\Omega(\bar{\lambda})$$

and

(S17) $$\Omega(\bar{\sigma})\approx\frac{k_BT_{eff}}{\omega(\bar{\sigma},T)},$$

where the entropic contribution is considered through an effective temperature $T_{eff}$, that satisfies

(S18) $$\frac{1}{T_{eff}}=\frac{1}{T}-\frac{1}{T_m}.$$

The temperature effect and the contribution of the activation entropy on the rate[2,3], is included in $\beta^*=(k_BT_{eff})^{-1}$.

We emphasize that the value of the activation volume is computed for a driving force that is the most probable one at the given temperature $\bar{\lambda}$, without any *a-priori*

assumptions on the dependence of the activation volume (or the activation energy) on the driving force.

**Note 2: The Relation between the Activation Parameters Calculated with Two Related Stress Components**

In the problem of nucleation-controlled plasticity, the CDF can be constructed either as function of the resolved shear stress on the slip plane $\tau$, or as a function of the compressive stress $\sigma$. Let us examine how the activation parameters calculated based on these two stresses are related. Both stress components are linearly proportional ($\tau = m\sigma$) and the analysis reported here can be generalized to any two different driving forces linearly related.

Since the difference between the CDFs, $F(\sigma)$ and $F(\tau)$, differs only in the scaled stress, the widths of the CDFs are scaled by the same factor

(S19) $$\omega_\tau = m\omega_\sigma .$$

Consequently, according to Eq. (S13), the activation volumes obtained from the different CDFs are related through

(S20) $$\Omega_\tau = \frac{k_B T_{eff}}{\omega_\tau} = \frac{k_B T_{eff}}{m\omega_\sigma} = \frac{\Omega_\sigma}{m} .$$

For clarity, we used in Eq. (S20) the stress-dependent part of the activation volume but the relation applies for the activation volumes since it relates the parameters at the same temperature. Since the activation volume is the derivative of the activation energy with respect to the corresponding driving force (either $\sigma$ or $\tau$) then

(S21) $$Q_\tau = \int \Omega_\tau d\tau = \int \frac{\Omega_\sigma}{m}(md\sigma) = \int \Omega_\sigma d\sigma = Q_\sigma .$$

Using Eq. (S16) to calculate the nucleation rate prefactor, with the relation found in Eqs. (S20) and (S21), yields the relation

(S22) $$\nu^0_{0,\tau} = \frac{\dot{\tau}}{N}\beta * \Omega_\tau\left(\bar{\tau}\right)e^{\beta Q_\tau(\bar{\tau})} = \frac{m\dot{\sigma}}{N}\beta * \frac{\Omega_\sigma\left(\bar{\sigma}\right)}{m}e^{\beta * Q_a(\bar{\sigma})} = \frac{\dot{\sigma}}{N}\beta * \Omega_\sigma\left(\bar{\sigma}\right)e^{\beta * Q_a(\bar{\sigma})} = \nu^0_{0,\sigma} .$$

To summarize the results, the CDF can be calculated either as a function of the compressive stress, which is the stress parameter accessible in the simulations, or the as a function of the resolved shear stress, which is the more natural variable for dislocation nucleation. If calculating the activation parameters according to the

former, the activation energy and entropy that corresponds to the resolved shear stress are equal, where the only difference is in the activation volume, which should be divided by *m*.

**Note 3: Fitting values for $T_m$ and $\nu_0^0$**

As demonstrated in the main text, a Gaussian function can be fitted to the distribution of nucleation stresses at a given strain rate. The standard deviation of the distribution $\omega$ is related to the activation volume at the most probable nucleation stress $\bar{\sigma}(T)$ based on Eq. (S13),

(S23) $$\Omega_G(\bar{\sigma},\mathrm{T}) \approx \frac{1}{\beta\omega(\bar{\sigma},\mathrm{T})},$$

While $\Omega_G(\bar{\sigma},\mathrm{T})$ is a function of both stress and temperature, the MD simulations provide values along certain couples of $T$ and $\bar{\sigma}(T)$. Under the assumptions described in Note 1, the results are fitted to the function

(S24) $$G(\sigma,T) = Q_0\left(1-\frac{\sigma}{\sigma_0}\right)^{\alpha}\left(1-\frac{T}{T_m}\right).$$

Since $T_m$ is unknown, a value is chosen and the calculated values of $\Omega_G(\sigma,\mathrm{T})$ are transformed into a set of values of $\Omega(\sigma)$, which are then fitted to

(S25) $$\frac{\Omega_G(\sigma,T)}{1-\dfrac{T}{T_m}} = \Omega(\sigma) = \frac{\alpha Q_0}{\sigma_0}\left(1-\frac{\sigma}{\sigma_0}\right)^{\alpha-1}.$$

The fit yields the values of $Q_0$ and $\alpha$ at given values of $T_m$.

Given that the energy barrier is known, the exact CDF can be calculated based on Eq. (S3),

(S26) $$F_{exact}(\sigma,T) = 1-\exp\left[-\frac{\mathrm{N}\nu_0^0}{E\dot{\varepsilon}}\int_0^{\sigma} e^{-\beta Q_0\left(1-\frac{\eta}{\sigma_0}\right)^{\alpha}\left(1-\frac{T}{T_m}\right)} d\eta\right].$$

$$= 1-\exp\left\langle -\frac{\mathrm{N}\nu_0^0\sigma_0}{E\dot{\varepsilon}\alpha(\beta^*\mathrm{Q}_0)^{1/\alpha}}\left\{\Gamma\left[\frac{1}{\alpha},\beta^*Q_0\left(1-\frac{\sigma}{\sigma_0}\right)^{\alpha}\right]-\Gamma\left[\frac{1}{\alpha},\beta^*Q_0\right]\right\}\right\rangle.$$

The value of $\nu_0^0$ in the CDF is also unknown. To fit the values of $T_m$ and $\nu_0^0$, a distance function is defined, for the difference between the exact CDF and the one

calculated from the MD simulations (using the Gaussian function), summed over all the temperatures chosen in the MD simulation

(S27) $$d(T_m,\nu_0^0)=\sum_{\{T\}}\int_0^{\eta}\left\langle F_{exact}(\eta,T)-\frac{1}{2}\left[1+erf\left(\frac{\eta-\bar{\sigma}(T)}{\sqrt{2}\omega(\bar{\sigma},T)}\right)\right]\right\rangle d\eta\,.$$

The distance as a function of both fitting parameters is shown in Fig. S2. The best fit (minimum in $d(T_m,\nu_0^0)$) is achieved with $T_m$=1650K and $\nu_0^0$=1.3·10$^{13}$ sec$^{-1}$. The dashed line indicates the best fit of $\nu_0^0$ for a given value of $T_m$. One can see that the fit is not very sensitive to the choice of $T_m$ for values above approximately 1500K. A similar insignificant sensitivity was pointed out by Chen *et al.*[1]. On the other hand, the value of $\nu_0^0$ has a significant effect on the accuracy of the fit and is of the order of 10$^{13}$ sec$^{-1}$. The fit for $T_m \lesssim$1500K becomes less accurate, and as we mention in the main text, at these values of $T_m$, only the MD results of the highest temperatures fit well.

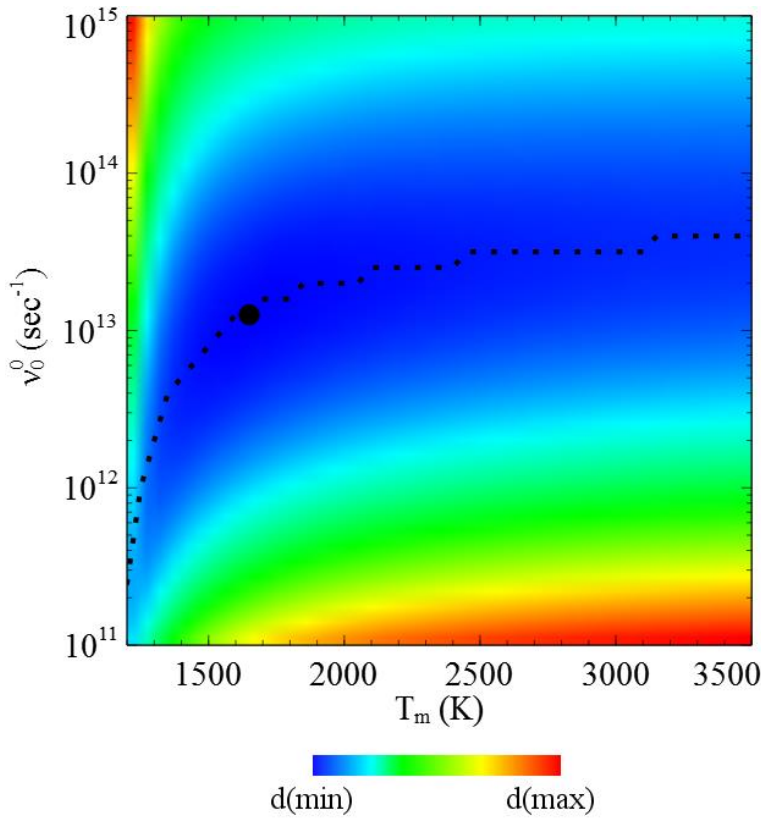


Figure S2: The distance between the calculated CDF and the MD simulations, as a function of $T_m$ and $\nu_0^0$. The dashed line indicates the best fit for a given $T_m$ and the black circle is the best fit within the whole range of $T_m$ and $\nu_0^0$ examined.

## References

1. Chen, L. Y., He, M., Shin, J., Richter, G. & Gianola, D. S. Measuring surface dislocation nucleation in defect-scarce nanostructures. *Nat. Mater.* **14,** 707–13 (2015).
2. Ryu, S., Kang, K. & Cai, W. Entropic effect on the rate of dislocation nucleation. *Proc. Natl. Acad. Sci. U. S. A.* **108,** 5174–8 (2011).
3. Ryu, S., Kang, K. & Cai, W. Predicting the dislocation nucleation rate as a function of temperature and stress. *J. Mater. Res.* **26,** 2335–2354 (2011).
